# Atrial Fibrillation Classification with Smart Wearables Using Short-Term Heart Rate Variability and Deep Convolutional Neural Networks

**DOI:** 10.3390/s21217233

**Published:** 2021-10-30

**Authors:** Jayroop Ramesh, Zahra Solatidehkordi, Raafat Aburukba, Assim Sagahyroon

**Affiliations:** Department of Computer Science and Engineering, American University of Sharjah, Sharjah P.O. Box 26666, United Arab Emirates; jramesh@aus.edu (J.R.); g00059068@aus.edu (Z.S.); asagahyroon@aus.edu (A.S.)

**Keywords:** biomedical informatics, cardiovascular disease, deep learning, ECG, heart rate variability, machine learning, PPG, smartphones, smart wearables

## Abstract

Atrial fibrillation (AF) is a type of cardiac arrhythmia affecting millions of people every year. This disease increases the likelihood of strokes, heart failure, and even death. While dedicated medical-grade electrocardiogram (ECG) devices can enable gold-standard analysis, these devices are expensive and require clinical settings. Recent advances in the capabilities of general-purpose smartphones and wearable technology equipped with photoplethysmography (PPG) sensors increase diagnostic accessibility for most populations. This work aims to develop a single model that can generalize AF classification across the modalities of ECG and PPG with a unified knowledge representation. This is enabled by approximating the transformation of signals obtained from low-cost wearable PPG sensors in terms of Pulse Rate Variability (PRV) to temporal Heart Rate Variability (HRV) features extracted from medical-grade ECG. This paper proposes a one-dimensional deep convolutional neural network that uses HRV-derived features for classifying 30-s heart rhythms as normal sinus rhythm or atrial fibrillation from both ECG and PPG-based sensors. The model is trained with three MIT-BIH ECG databases and is assessed on a dataset of unseen PPG signals acquired from wrist-worn wearable devices through transfer learning. The model achieved the aggregate binary classification performance measures of accuracy: 95.50%, sensitivity: 94.50%, and specificity: 96.00% across a five-fold cross-validation strategy on the ECG datasets. It also achieved 95.10% accuracy, 94.60% sensitivity, 95.20% specificity on an unseen PPG dataset. The results show considerable promise towards seamless adaptation of gold-standard ECG trained models for non-ambulatory AF detection with consumer wearable devices through HRV-based knowledge transfer.

## 1. Introduction

Cardiovascular diseases (CVD) are the leading cause of death worldwide, with the World Health Organization (WHO) in 2016 estimated 17.9 million deaths annually [1]. CVD is a group of conditions that affect the heart’s rhythm mechanical function, and electrical activity [2]. This is associated with an increased likelihood of strokes and heart failure. Timely detection through regular monitoring of CVD is necessary to improve the treatment process for heart conditions and lower the risk of mortality [3]. Cardiac arrhythmia is categorized under CVD and is characterized by the disordered electrical activity of the heart. An arrhythmia can manifest as irregularly rapid heart rhythms (tachycardia) or anomalous slow heart rhythms (bradycardia). AF is one of the most common types of cardiac arrhythmia. In this work, the focus is on the classification of (i) normal sinus rhythm (NSR), and (ii) atrial fibrillation (AF). Goldberger et al. [4] defines NSR as a rhythm with normal (1:1) atrioventricular conduction and a normal PR interval (the interval between atrial depolarization and ventricular depolarization) at a heart rate between 60 and 100 beats/min, although normal heart rates may vary between individuals. The work reported in [5] defines AF as an arrhythmia with uncoordinated atrial activation and characteristics of irregular beat-to-beat intervals, absence of repeating P waves (indicates atrial depolarization), and irregular atrial activity.

The common technique for the clinical diagnosis of cardiac arrhythmia is based on the electrocardiogram (ECG). The ECG is a test that uses skin level electrodes with built-in sensors to measure the heart’s electrical activity and identify abnormal heart rhythms and additional pathological conditions [6]. However, despite the multi-faceted diagnostic nature of ECG, most dedicated ECG devices available currently are expensive and are typically used within clinical or limited ambulatory settings [7]. While wearable ECG devices are emerging commercially, individuals gravitate towards smart wearables that can serve general functions and are not only intended for health monitoring. Off-the-shelf smartphones and wearable devices that use photoplethysmography (PPG) sensors can serve as an affordable alternative to existing ECG devices, albeit as a supplementary approach for screening and not for conclusive diagnosis. PPG sensors are optical light sensors that record blood volume variations at sensitive peripheral sites of the human body, such as fingertips, wrist, and earlobes [8]. Moreover, PPG sensors are currently used extensively by fitness tracking applications to estimate the physiological events of heart rate and heart rate variability (HRV) [9]. PPG signals differ morphologically from ECG signals but exhibit similar characteristics as the HRV. This is termed as pulse rate variability (PRV). The advantages of PPG sensor-based consumer devices for cardiac arrhythmia monitoring are that they are relatively less obtrusive than their ECG counterparts. Their ubiquitous nature facilitates higher adoption by the general population. Despite these advantages, PPG recordings are more susceptible to noise saturation and variations in signal quality caused by user movement and skin tones [10].

The data features extracted from ECG and PPG heart signals to develop learning algorithms can be categorized as temporal or morphological [11]. Temporal features are the time-domain metrics such as the time between heartbeats. Many deep learning works in this area pursue the development of morphology-based models using the PPG segments or corresponding images to leverage the robustness and have generally superior performance in classification problems. However, there are significant challenges in developing PPG-based analytical models due to the limited public availability of universally reviewed benchmark databases, as opposed to the abundant ECG signals databases. Moreover, signal quality and noise saturation can corrupt the performance of the developed models. The manual annotation process for creating labeled datasets is complex and has the consistency issue of interrater variability [3]. Interrater variability arises when multiple expert annotators are involved in labeling heart rhythms manually. Different labels are assigned to the same data instance due to differences in their specific experiences. In practice, it is difficult to reach an agreement across multiple experts if the data are not ideally preprocessed and motion artifacts are not eliminated. This is the case with the PPG signal annotation efforts in most of the literature. Moreover, most developed algorithms in the literature are only applicable in controlled clinical settings, which hinders early prognosis accessibility to the general population.

Although there are inherent morphological differences between ECG and PPG-based signals, the studies reported in [12,13] have exhibited a high degree of correlation between the signals, especially their corresponding temporal HRV features. HRV measures the variation in terms of time between consecutive instantaneous heartbeats, measured through the ECG [9].

The PRV and HRV parameters, derived from ECG and PPG, respectively, exhibit similar properties under certain conditions. The properties have higher levels of agreement/equivalence when the PPG signals are not excessively situated with motion artifacts. Various predictive and detection models have been implemented using different HRV metrics with standard statistical and machine learning approaches [14,15,16,17,18,19]. However, there are considerably fewer deep learning-based models oriented towards usage in smartphones and wearable devices. Deep learning has recently emerged as an effective methodology for cardiac classification tasks [20]. The experiments reported in [21,22,23,24] have achieved successful ECG signal classification using ECG databases by implementing convolutional neural networks. However, the existing approaches are designed for use in controlled hospital settings.

This research addresses the scarcity of publicly available PPG datasets, limited reproducible approaches in the existing literature, and varying sensor specifications. This work proposes implementing a deep learning approach that utilizes the knowledge transfer paradigm for cross-domain generalizability by training a model on ECG databases and adapting the developed model for PPG signals-based AF classification. The commonality in the distribution of temporal features derived from HRV (ECG) and PRV (PPG) is leveraged as input features to implement a one-dimensional convolutional neural network for classifying NSR and AF rhythms. The motivation for this approach is to introduce generalizable deep learning models that can mitigate the challenges associated with purely PPG- based analytical models and facilitate close to real-time AF detection.

The contributions of this work are as follows:

- The incorporation of the state-of-the-art methods for ECG and PPG signal processing and HRV feature extraction from short length signals;
- The development of a deep learning model trained on HRV features derived from on gold standard ECG for classification of AF with PRV derived from PPG features through transfer learning;
- The evaluation of the developed model performance on three ECG datasets and a PPG dataset composed of wrist-worn wearable signals which achieved competitive results when compared to the recent literature;
- The implementation of a cloud-based platform and the evaluation of the developed model performance on PPG signals acquired from live subjects via smartphones.

This paper is organized as follows: Section 2 introduces the background of the concepts used in the analysis of this work, Section 3 details the proposed approach, Section 4 presents the obtained results of the model, Section 5 discusses the results, and is followed by the conclusion and future work in Section 6.

## 2. Background 

### 2.1. Heart Activity Measures 

**Figure 1:**
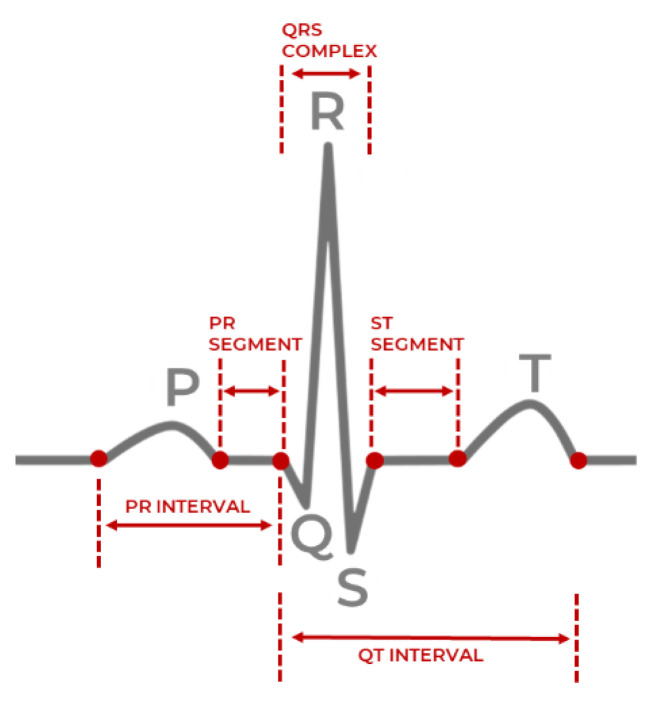
Single heartbeat sample with the QRS complex [4].

The entire sequence of a single heartbeat, beginning with the initial atrial excitation and concluding with the exit from the ventricular chambers, is called PQRST and is shown in Figure 1. An electrical impulse travels through the heart during each heartbeat, causing the heart muscles to pump blood. After a flat line driven by the impulse traveling to the bottom heart chambers, the right and left atria (upper heart chambers) create the first wave, called P wave. The right and left ventricles (bottom chambers) make the next wave called the QRS complex, and the final T wave indicates the repolarization of the ventricles. The QRS complex is the peak shown in Figure 1. Variations in parameters obtained from ECG and PPG, such as the duration and rate of heartbeats, can help detect abnormal heart activity [6].

**Figure 2:**
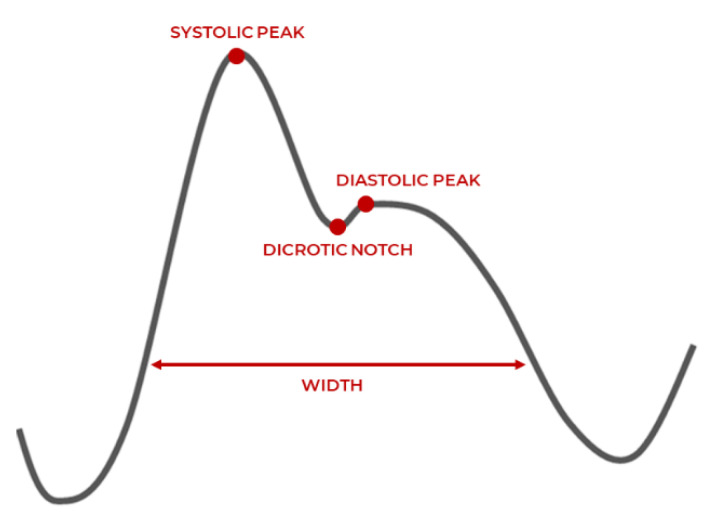
PPG waveform characteristics [3].

PPG is an optical light-based technique to measure the volumetric change of the heart. As the heart contracts, blood pressure in the left ventricle (bottom chambers) increases. This is reflected by an increased pressurized pulse of blood into the capillaries and arteries of the body, indicated by discoloration of the skin. An LED light measures the difference in the amount of light reflected from sensitive areas, where the arteries are close to the skin, such as fingertips or earlobes, which is then used to measure an individual’s heart rate [25]. A typical waveform of the PPG signal and its characteristic parameters are shown in Figure 2, which are the systolic peak, pulse with and diastolic peak, and dicrotic notch. Smartphones and wearable devices are generally accurate in acquiring PPG signals when the user is at rest, but potential inaccuracies are introduced because of motion artifacts and diverse skin tones. Motion artifacts typically occur due to misplacement of sensors such that it does not make sufficient contact with the measurement site. Various skin tones affect the reflective properties of the optical light differently and therefore affect the accurate assessing of the changes in blood volume under the skin [26].

PPG has two peaks corresponding to the blood volume changes in the microvascular bed of tissue around the physical measurement site of the fingertips, earlobes, wrists, etc. Systolic peak is caused by the direct pressure wave traveling from the left ventricle to the body periphery (heart contraction). The diastolic peak reflects the pressure wave by arteries in the lower body (heart relaxation). The pulse width correlates with systemic vascular resistance, and the dicrotic notch reflects a transient increase in aortic pressure [27]. Although PPG is an indirect way to record the heart’s activity, it has a high correlation with ECG signals. Its portability and relatively inexpensiveness make it a valuable alternative method to monitor cardiac activity [8].

### 2.2. Heartrate Variability 

The HRV phenomenon is controlled by the Autonomous Nervous System (ANS) and is a direct result of the behavior of the primitive part: the parasympathetic nervous system. The brain processes information in the hypothalamus region, and the ANS sends signals to the rest of the body to either stimulate or relax different functions. Auto-responses from the ANS are elicited in the event of stress, fragmented sleep, unhealthy diets and other chemical or neural factors affecting a person’s resting state. HRV is a non-invasive way to identify ANS imbalances, as when the nervous system is behaving unusually, the variation in the heartbeats is relatively more erratic. A higher HRV score generally indicates better cardiovascular fitness and resilience to stress. In comparison, a lower HRV score is associated with an increased risk of cardiovascular health and mental health concerns [9].

The primary feature used in HRV calculations is the time between each successive heartbeats, or the time between successive normal or abnormal QRS complexes/peaks in milliseconds, defined as the R-R peak interval. Estimation of the R-R interval involves first detecting the QRS complexes/peaks and subtracting the observed times of successive peaks. It should be noted that a distinction is made between R-R intervals, and the typically synonymous N-N interval, as the latter only accounts for normal-normal beats, while the former accounts for normal-normal, normal-abnormal, or abnormal-abnormal cases. 

PRV is used to measure the similar inter-beat variation property with PPG signals, and this denotes the pulse-to-pulse variation in time. PRV quantifies approximately the same behavior as the intervals between successive R peaks or QRS complex observed in ECG with the systolic peak-to-systolic peak or diastolic peak-to-diastolic peak intervals.

Malik et al. [28] observed the potential of HRV in assessing the role of ANS fluctuations in normal healthy individuals and those with diseases. Relevant measures were selected from the previous research and used as HRV features for the scope of this work.

This work primarily uses the formulas shown by Equations (1) and (2) to calculate Root Mean Square of Successive Differences between the R-R intervals (rMSSD) [28] and Standard Deviation of RR intervals (SDRR) [28]:

(1) $$rMSSD=\sqrt{\frac{\sum_{i=1}^{N-1}{\left(RR_{i}-RR_{i+1}\right)}^{2}}{N-1}}$$

From Equation (1), N is the number of R-R intervals and $RR_{i}$ is the location of the *i*th QRS complex/peak observed at a time in milliseconds.

(2) $$SDRR=\sqrt{\frac{1}{N-1}*\overset{N}{\underset{j=1}{\sum }}{\left(RR_{j}-\bar{RR}\right)}^{2}}$$

From Equation (2), N is the number of R-R intervals, $RR_{j}$ is the location of the *j*th QRS complex/peak observed at a time in milliseconds. 

The features of rMSSD and SDRR respectively reflect the number of fluctuations in heart rhythms and the degree of variation between heart beats. Hence, both are vital features to consider when aiming to predict the cardiovascular state. Various cardiac conditions were detected using short-term HRV features, with rMSSD, SDRR, and pRR50 being the most useful in predicting changes in parasympathetic activity and even being a possible indicator of cardiac mortality [29]. Additional HRV features are also included in Table 1 and used in this work. Those additional features include the coefficient of variation in R-R intervals (CVRR) and coefficient of variation in the differences of successive R-R intervals (CVSD), as they are features that improve the classification of CVD [30]. Researchers recorded PPG signals from the fingertips of subjects extracted PRV features, such as rMSSD, SDRR, and pRR50, compared them with the same features obtained from ECG to validate the accuracy, and found that the average error rate was less than 6% [30]. Another study used wearables to compare the time domain features (rMSSD, SDRR) of HRV extracted from ECG and PRV extracted from PPG signals and found that PPG signals can be used as an alternative source for HRV measurement [31]. The features used in this work are presented in Table 1.

This relationship can be utilized to monitor individuals’ cardiovascular health with off-the-shelf sensors for classifications and early detection of diseases. The commonality between the behavior of the HRV and PRV parameters can be utilized to enable generalized detection of AF across two different modalities: ECG and PPG. The model is trained on HRV features derived from ECG signals within the three ambulatory datasets. The model is tested and finetuned on PRV features derived from PPG signals within the wearable dataset.

For verification of the created dataset and its respective HRV values for different R-R interval measures, it was compared to the short-term normative values reported in [32], and the reference ranges for HRV from ECG recordings [33]. The HRV features of NN intervals, rMSSD, and SDRR were the most reported along with their normative ranges, and it is shown in Table 2. This comparison ensured that the extracted PRV features for real-time samples from the low-cost PPG sensors from wearables were within reasonable bounds of the ground truth cases and should remain valid for this experimentation.

## 3. Proposed Approach

**Figure 3:**
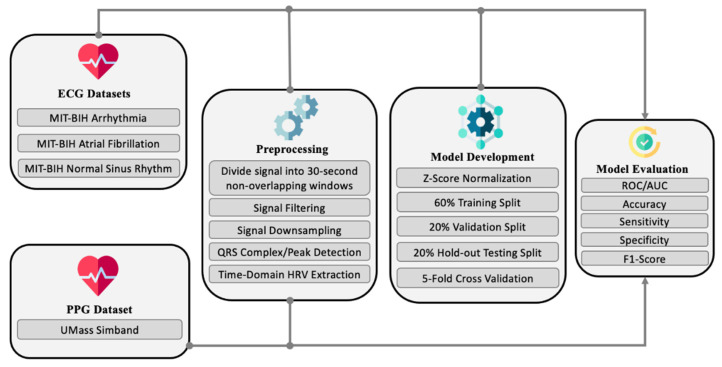
High-level view of the proposed approach.

The proposed approach has three main stages after the initial acquisition of datasets, as shown in Figure 3. The first stage involves preprocessing the signals in terms of filtering, peak detection, and feature extraction. The second involves the one-dimensional convolutional neural network (CNN) model development for binary classification between NSR and AF with temporal HRV features, and trained with the ECG datasets. The third stage involves model evaluation. The model evaluation is done on both the holdout testing on a subset from the ECG datasets and out-of-sample cross-domain testing instances from the PPG datasets. Each stage is detailed in the following subsections.

### 3.1. ECG Datasets

NSR and AF rhythms are collected from three datasets [34]: MIT-BIH Normal Sinus Rhythm (NSR-DB), MIT-BIH Atrial Fibrillation (AF-DB), and MIT-BIH Arrhythmia (ARR-DB).

Using ambulatory ECG recorders, each record was acquired from patients referred to the Arrhythmia Laboratory at the Beth Israel Deaconess Medical Center, Massachusetts Institute of Technology. They are accessible via the Physiobank repository, a digital archive of well-characterized biomedical signals created by the United States National Institutes of Health for use by the research community [35].

AF-DB consists of 23 two-channel ECG recordings (sampled at 250 Hz), from subjects with paroxysmal atrial fibrillation, atrial flutter, AV junctional rhythm, and normal rhythms, with a typical recording bandwidth of approximately 0.1 to 40 Hz. NSR-DB consists of 18 two-channel ECG recordings (sampled at 128 Hz) from subjects with no significant arrhythmia or heart abnormalities. ARR-DB consists of 48 records, each containing two-channel ambulatory ECG signals of 30-min duration. Lead 1 channel ECG signals, which record the right ventricle and right atrium, are used in this work.

The signals in AF-DB have rhythm annotations indicating NSR and AF. Meanwhile, the signals in NSR-DB and ARR-DB have heartbeat annotations as well, in addition to rhythm annotations for AF and NSR. The annotations are provided in terms of a distinct beginning and end label pertaining to particular regions of the signals. The heartbeats in NSR-DB and ARR-DB follow the recommended standards of the Association for the Advancement of Medical Instrumentation [36]. Hence, the annotations/labels for each heartbeat in the NSR-DB and ARR-DB fall into multiple categories [37]. The beat superclasses and their corresponding beat annotations of interest in this work are N: (*N*, *L*, *R*, *B*) and S: *A*, *a*, *J*, *S*, *j*, *e*, *n*. While the primary focus is on heart rhythm classification, specific samples in the dataset are considered on a heartbeat segment basis for incorporating cases of atrial premature complexes (APC) [38]. The rationale for incorporating heart rhythms with high saturation levels of anomalous heartbeats is to contribute stochasticity (diversity) to the AF class. The expectation is that the dataset consisting of contiguous AF rhythms and AF rhythms interspersed with normal and other types of beats will allow for the eventual detection of varying anomalous rhythms that differ considerably from the purely NSR training samples [39,40]. 

### 3.2. PPG Dataset

The privately held UMass PPG database (UMass-DB) [41] collected by the University of Massachusetts Medical School was used for further testing to discover the strengths and weaknesses of the model. The authors of [42] granted access to this dataset and consists of 37 subjects, with 10 having AF. The PPG signals were recorded at a sampling frequency of 128 Hz from the Simband, smart wristwatch provided by Samsung, which has 8 PPG sensors, a triaxial accelerometer, an ECG lead, and a temperature sensor [42].

**Figure 4:**
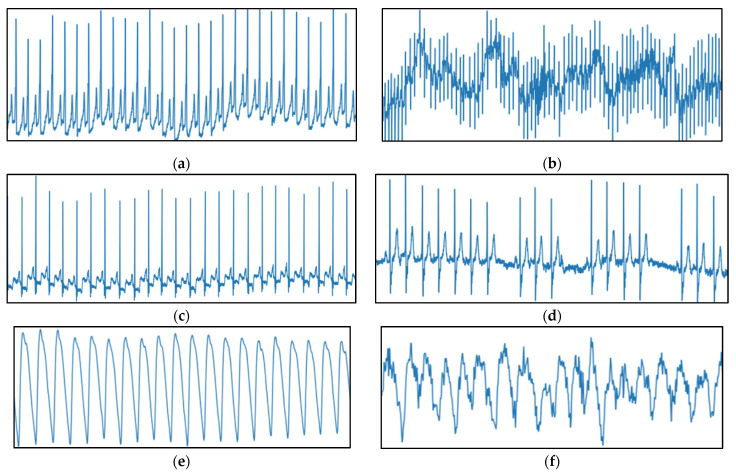
Sample 30-s heart rhythm instances represented as raw amplitude (y-axis) against time (x-axis) from ECG datasets; (**a**) NSR ECG from AF-DB (Patient 4015); (**b**) AF ECG from AF-DB (Patient 4043); (**c**) NSR ECG from ARR-DB (Patient 100); (**d**) AF ECG from ARR-DB (Patient 222) and PPG dataset; (**e**) NSR PPG (Patient 4002); (**f**) AF PPG (Patient 4012).

Figure 4 presents the typical characteristic heart rate rhythm samples from both datasets reflecting NSR and AF, respectively, across the ECG and PPG modalities. As observed from Figure 4a,c,e, NSR instance is a normal heart rhythm that maintains a steady rate with no irregularities. From Figure 4b,d,f, the AF instance is a sustained unsteady heart rhythm with rapid fluctuations. 

### 3.3. Preprocessing 

Initially, the signals with rhythm annotations of NSR and AF from AF-DB, ARR-DB, and NSR-DB were divided into 30-s samples with no-overlapping windows. The segmented 30-s signals retained the respective label of NSR or AF as multiple 30-s samples can be obtained from a single longer signal with the same annotation. In the case of ARR-DB, all signals with annotations corresponding to non-atrial complications, such as paced rhythms, ventricular bigeminy, trigeminy, tachycardia, were ignored. 

Most AF contiguous data samples originated from the AF-DB, with approximately 3.6% being from the ARR-DB dataset. From the NSR database, 15% of the total NSR rhythm records were arbitrarily selected. Most NSR data originated from NSR-DB, followed by ARR-DB while AF-DB contributed only 5% of the total NSR samples. All the signals accounted for had the highest resolution in terms of QRS complex certainty. 

In addition, signals with ARR-DB were examined further in terms of heartbeat saturation to determine the presence of excessive supraventricular activity, which is associated with an increased risk of developing atrial fibrillation [43]. The examined signals were annotated with APC, supraventricular tachyarrhythmia (SVTA), atrial couplets, or atrial flutter. As per AAMI standards, all considered heartbeats in the 30-s window derived from these signals belonged to the class N or S. The beats denoted by S can be referred to as supraventricular ectopic beats or premature beats. Although ectopic beats are mostly harmless, recent studies have shown that frequent repetitions of supraventricular ectopic behavior can indicate the presence of potential atrial abnormalities [44].

The criteria for judging the label of a 30-s rhythm are based on the saturation level of class S beats. If zero S beats are present, then it is ignored, and if over 50% of the beats are S with an annotation of a, *J*, *A*, *S*, *j*, *e*, or *n*, it is treated as an AF rhythm. The passage from heartbeat types to heart rhythms is not necessarily direct. Thus, this rule is to ensure that only segments consisting of non-isolated beats are treated as AF samples. 

Individuals in real scenarios may not always exhibit signs of sustained arrhythmia. It is possible for a fluctuating pattern between normal rhythms, where relatively shorter (<30 s) intermittent periods of abnormal heart behavior associated with AF can be observed, and thereby contributing to AF risk stratification. Excessive ectopic activity can cause palpitations, light-headedness, and increased awareness of heartbeats [45]. For instance, patient 232 does not have any AF rhythm annotations, but has frequent ectopic runs. The cardiologists’ notes associated with the annotated record of patient 232 report the presence of sick sinus syndrome, which is an abnormality in the right atrium of the heart. To address this case of potential variability in patients and boost the robustness in classification performance of the developed model, instances that are not solely NSR but anomalous to a considerable degree were treated as an AF class instance. 

As per the findings of [27,46], a second-order Butterworth filter was applied with the bandpass frequencies of 8Hz–20Hz for removing baseline drift, motion artifacts and minimizing other ECG features such as the P and T waves. The signals of the MIT-BIH Arrhythmia, MIT-BIH NSR, and MIT-BIH AF databases have sampling rates of 360 Hz, 128 Hz, 250 Hz, respectively. Fast Fourier (FFT) resampling is applied to down-sample the signals to 50 Hz, as the signals from the three MIT-BIH databases have different original sampling rates. It, therefore, must have the same frequency before any further processing. The method reported in [46] achieves the highest signal-to-noise ratio and optimal QRS complex detection on the MIT-BIH databases instead of techniques such as the Pan Tompkins algorithm [47], and the former method is utilized to produce a list of the peaks necessary to derive the time-domain HRV features.

PPG signal filtering was conducted with a 3rd order Butterworth filter with 0.5 Hz and 8 Hz cutoffs to remove powerline interference, motion artifacts, and other saturated noise [48]. The UMass dataset signals were down-sampled from 128 Hz to 50 Hz using FFT resampling, similar to the approach executed in [42]. Systolic peak detection in the PPG signals utilized the algorithm outlined in [49], where two event-related moving averages with an offset threshold empirically yielded higher accuracy than the alternative techniques of Billauer [50], Li [48], and Zong [51]. 

The decision for down-sampling all signals to 50 Hz, instead of up-sampling any acquired signals to 128 Hz is based on two key factors. Firstly, most PPG based devices do not have a high sampling rate (~128 Hz), and vary from 60 Hz to 100 Hz based on the quality of the sensor and the battery levels of the device the sensor is embedded in. However, the minimum sampling frequency required is 50 Hz to derive reasonably accurate HRV and PRV parameters with a low margin of error from ECG and PPG signals, respectively [52,53]. Secondly, the computational overhead is reduced without a significant effect on the signal acquisition or processing aspects, which can extend the deployment of the proposed model in this work to resource-constrained wearable devices. 

It is to be noted that the systolic peak detection algorithm for PPG signals proposed in [48] is a modified variant of the QRS peak detection algorithm for MIT-BIH database ECG signals proposed in [46]. This work performed filtering as per the recommended cutoff frequencies before applying the algorithm, as mentioned previously in this section. The general description of the algorithm reported in [46,48] is as follows:

(i) Consider a filtered signal S[n], consisting of a sequence of n samples over a sampling period T=30 s, as input to either the ECG variant of the algorithm or the PPG variant of the algorithm;
(ii) Detect R peaks in the ECG signals and systolic peaks in the PPG signals through a combination of potential block generation and thresholding;
(iii) Preprocess PPG systolic peak detection (step skipped for ECG R peak detection in the squaring phase), where large differences resulting from the systolic peak are emphasized, while the small differences caused by the diastolic peak, dicrotic notch, and saturated noise are suppressed;
(iv) In the potential block generation phase, regions of the signal S[n] where peaks are likely to occur are demarcated in terms of the onset and offset points by two moving averages $MA_{peak}$ and $MA_{beat}$;
(v) $MA_{peak}$ estimates the possible regions of R peak or systolic peak amplitude and $MA_{beat}\;$represents the amplitude in regions of a full heartbeat (RR peak, or systolic peak-to-systolic peak);
(vi) The window size $W_{1}$ of the $MA_{peak}$ is selected based on a healthy adult’s average duration of a QRS complex (100 milliseconds) or systolic peak (111 milliseconds) depending on the signal modality. The window size $W_{2}$ for the $MA_{beat}$ is selected based on the average duration of one full heartbeat (525 ms) or systolic peak (667 ms) in a healthy adult [49]. The defined windows $W_{1}\;and$ $W_{2}$ bound the lower limit $TH_{1}$ and upper limits of the generated blocks, respectively;
(vii) The specific windowed regions where the amplitude values of $MA_{peak}$ are greater than $MA_{beat}$, are selected as blocks of interest;
(viii) As a signal S[n] can be saturated with noise and motion artifacts during acquisition, the thresholding phase eliminates blocks that are likely to hinder accurate peak detection. The threshold α specifies the anticipated width of a block, and any detected QRS complex or systolic peaks with width less than this threshold is rejected. An optional parameter β can be added to the threshold to consider minor deviations in peak width and either tighten or loosen the constraints on a rejected block;
(ix) The output of the algorithm is a list of peak locations and their corresponding times in milliseconds.

After performing the peak detection algorithm summarized in Algorithm 1, a list of peak locations and their occurrence times enables the estimation of RR intervals or systolic peak-to-systolic peak intervals. From the intervals, the time-domain HRV and PRV features are derived in terms of their statistical characteristics as described in Table 1.
**Algorithm 1.** Pseudocode of peak detection algorithm and feature detection for dataset ***D***.**FOR**$x_{i}\;$in ***D*** (ECG or PPG data instance from dataset, where i = {0…size(D))Filtered signal S[n] = BandpassFilter($x_{i})$Let peaklist={} (Peak amplitudes)Let timelist={} (Peak times)Let BlocksOfInterest={}Let$\;y_{i}=\{\}$Set $W_{1}$ = Average ECG or PPG peak durationSet $W_{2}$ = Average ECG or PPG beat durationSet $MA_{peak}$ = MovingAverage(S[n], $W_{1}$)Set $MA_{beat}$ = MovingAverage(S[n], $W_{2}$)Set threshold α = $W_{1}+\beta$**FOR** n = 1 to length($MA_{peak}$)**IF** $MA_{peak}\left[n\right]$ > $MA_{beat}\left[n\right]$ **THEN**      BlocksOfInterest[n]=1**ELSE**      BlocksOfInterest[n]=0**END IF****END FOR****FOR** j = 0 to length(BlocksOfInterest)**IF** width(BlocksOfInterest[j]) ≥ α **THEN**peaklist[j] = max(BlockOfInterest[j])timelist[j] = time(BlockOfInterest[j])**ELSE**;       reject block**END IF****END FOR**{rMSSD, …,pRR50}= Calculate HRV/PRV (peaklist, timelist)Transformed data instance $\;y_{i}$ = {rMSSD, …,pRR50}Save $y_{i}\;$to updated dataset $\bar{D}$**END FOR**

Finally, Z-score normalization is performed on the derived features. All ECG and PPG datasets signal instances are fixed with zero mean (µ = 0), and unit standard deviation (σ = 1.) This step mitigates amplitude scaling issues, offset effects, and reduces drastic variability in the signal values. Table 3 presents total data samples of NSR and AF classes after pre-processing. 

### 3.4. Model Development 

**Figure 5:**
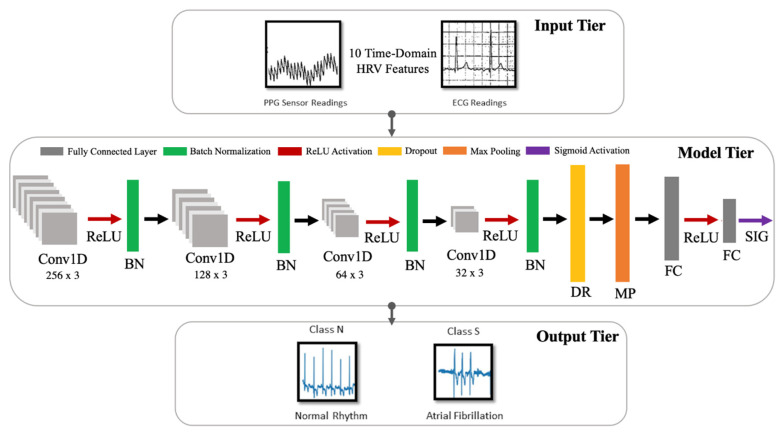
Architecture for the proposed CNN model.

The model developed in this work is a one-dimensional 12-layer CNN for the classification of NSR and AF. The proposed architecture for the CNN is depicted in Figure 5, outlining the input tier, model tier, and output tier. The model receives temporal HRV features extracted from ECG signals as input, propagates them through the neural network, and outputs a single output indicating whether the input instance belongs to NSR or AF class. A detailed summary of the CNN properties and parameters is listed in Table 4. The configuration of the layers and their respective parameters reported were attained after hyperparameter tuning through GridSearch. 

A single model is selected after training and evaluation. It is trained and tested using the HRV features derived from ECG, and finetuned to classify AF with PRV features derived from PPG. Due to the inherent similarities between the statistical properties of HRV and PRV, this approximation makes it possible for a unified AF representation across two wearable modalities.

There are three types of layers within a CNN: convolutional, pooling, and fully connected layers. An instantiated convolutional layer detects local conjunctions of features from a preceding layer which can be either an input layer or another convolutional layer. The convolutional layer merges semantically similar input features into a single learned representation. It is to be noted that features in the context of the neural network imply semantic similarities or overarching patterns detected across the provided inputs (a unified vector of HRV features). Receptive fields in each convolutional layer focus on different aspects of the derived features to create their internal representation of the inputs. The property of shared weights ensures that general features common to all data samples are learned once and shared with the other convolutional layers in the network. Subsampling reduces the dimensionality of the data to identify the most significant features. This can be related to size (spatial) or time sequence (temporal). A set of weighted vectors known as a filter/kernel outputs feature maps based on local receptive fields at each layer. These feature maps usually hold general characteristic information inferred from input feature data samples at a particular layer by the neural network [54].

Each layer of the proposed CNN architecture and the components of activation and regularization presented in Figure 5 are described as follows:

1. Convolutional Layer (Conv1D): In this layer, a convolution operation using Equation (3) is performed by sliding the filter/kernel over the input features to obtain a feature map as the output.
  (3) $$c_{m}=\overset{N-1}{\underset{n=0}{\sum }}f_{n}k_{m-n}$$
  From Equation (3), *k*, *c*, *f*, and *N* denote the inputs, filter/kernel, the output feature map, and the number of elements in input *k*, respectively. In the CNN model developed for this work, there are four convolutional layers with 256, 128, 64 and 32 filters, respectively. The filter dimensions used in this layer are 5 × 5, which yielded the best result.
2. Fully Connected Layer (FC): This layer compiles the results obtained from the preceding convolution and pooling layers to estimate an output classification label using Equation (4) [55]:
  (4) $$x_{i}=\underset{j}{\sum }w_{ji}y_{j}+b_{i}$$
  From Equation (4), *w* and *b* denote weights and biases, respectively. Here, *y* is the output from a previous layer *j* and *x* is the output of the current layer *i*. In the CNN model developed in this work, there are two fully connected layers, with 8 and 1 neurons, respectively.
3. Pooling Layer (MaxPooling1D): In this layer, the maxpooling operation is a type of spatial sub-sampling method that decreases the size of the feature maps derived by the convolutional layers. This is performed to retain only the features contributing significantly to the internal knowledge representation of the CNN, which is learned through the training process. In the CNN model developed for this work, there is 1 pooling layer, with 32 filters after the final convolutional layer and the following dropout layer. The filter dimensions of the pooling size used in this layer are 2 × 2.
4. Activation Functions: This determines the firing threshold of neurons in the hidden layer based on the weighted sum of input and biases.
  - Rectified Linear Unit (ReLU) [56]: This is the activation function that is used in all three convolutional layers of the network. The Rectified Linear Unit produces 0, as an output x<0, and then produces a linear output with slope 1, when x>0. It introduces non-linearity and mitigates the vanishing gradient problem, which is where the lower layers of the network train slowly as the gradient of optimization decreases exponentially. This leads to sparse neuron activation, more straightforward output, and makes computations easier while preserving the significant receptive fields of the convolution layers.
  - Sigmoid [57]: An activation function used in the second fully connected layer, with 1 neuron. Sigmoid activation functions are monotonic and differentiable. Their mathematical property maps real number values to the [0, 1] range to render the output as a probability, given the particular set of transformed input HRV features. In this work, the binary classification output of 0 indicates that an instance belongs to the NSR class, and 1 means that it belongs to the AF class.
5. Regularization [58]: This is a technique to prevent overfitting. Overfitting limits the ability of the model to predict new data, which means the network has learned only the specific features of the training set, like memorization, and cannot perform generalization on similar data. To mitigate this, the following two methods were used after all four convolutional layers.
  - Batch Normalization (BN) [59]: This technique reduces the covariance shift, meaning that minor features differences that do not contribute heavily to the overall model performance will not be considered with high priority. Therefore, minor changes between the ranges of training data, validation data, or unseen data will not affect the classification performance and allow each layer to be more independent about certain input features.
  - Dropout (DP) [60]: This technique randomly drops neurons and their connections to prevent neurons from co-adapting. This makes each neuron more responsible for capturing the overall data representation and contributing to the final output. The dropout rate, which reflects the percentage of random neurons to be dropped, was set to 0.2.

### 3.5. Training and Testing 

The CNN model is trained with the back-propagation algorithm [54] with a mini-batch of 16. According to [61], taking a subset of the entire data for each epoch improved generalization performance and had a smaller memory footprint. An epoch is the number of times the training set passes through a neural network completing a feed-forward and back-propagation phase. In this work, the total number of epochs was 50. The Adaptive Moment Estimation (ADAM) [62] optimizer was used for effective training convergence.

From the dataset, 80% was randomly divided for training and validation, and 20% was used as the test set. The Stratified k-fold cross-validation strategy was implemented with k = 5 [63]. In each fold, the training and validation subset is randomly divided into 5 equal parts, where with cross-validation, each data instance is used for both training and validation. Stratified k-fold cross-validation ensures that the class distribution in each of the five equal parts remains consistent across iterations to address potential biases. This was conducted to observe the generalizability and variability of the developed model to reflect its performance with new data. The 20% testing subset serves as the holdout data that the model has not been trained/validated with.

## 4. Results

This section describes the environment setting, reports the achieved diagnostic performance measures of the proposed convolutional model neural network on the ECG training data and unseen PPG data. To assess the implementation feasibility of the developed model, it was interfaced with a smartphone application and integrated within a health monitoring context.

### 4.1. Implementation Environment 

The proposed CNN algorithm was implemented on a workstation with Windows OS, an Intel Kabylake 2.80GHz processor (i7-7700HQ), and 16 GB of RAM. The time required for training and testing the CNN model with 50 epochs was approximately 4420.67 s. The deep learning platform employed in this work was Keras [64], a high-level neural networks framework with a Tensorflow backend [65]. The Waveform-Database Package (WFDB) published by Physionet was used to directly access the MIT-BIH Arrhythmia dataset [35], consisting of heart rhythm samples and their respective annotations. The Sklearn module was used for data preprocessing and normalization operations [66]. Neurokit (NK), a toolbox for statistics and neurophysiological signal processing, was used to extract the ECG and PPG time-series features [67]. 

### 4.2. Model Evaluation on ECG Datasets 

The diagnostic performance measures of accuracy, sensitivity, specificity, F1-score, and AUC are evaluated on a holdout test set in each of the five folds. Accuracy is the proportion of true outputs with respect to all data instances. Sensitivity is the model’s ability to classify data instances belonging to a certain class correctly. Specificity is the model’s ability to correctly distinguish data instances that do not belong to specific classes. F1-score is the harmonic mean between precision (ratio of correctly distinguished positives over all predicted positive) and recall (sensitivity), and the area under the curve (AUC) measures the quality of binary classification outputs in terms of sensitivity against false positive rate. To develop high-fidelity biomedical models as the proposed approach, high sensitivity and specificity are vital. They gauge the model’s ability to correctly detect patients with a certain cardiac arrhythmia and correctly detect patients without cardiac arrythmia [68].

To calculate the measures as in Equation (5), the model classification outputs must be quantified in terms of True Positives (TP), False Positives (FP), False Negatives (FN) and True Negatives (TN) [69].

(5) $$\begin{array}{c} Accuracy=\frac{TP_{NSR}+TN_{NSR}}{TP_{NSR}+TN_{NSR}+FP_{NSR}+FN_{NSR}}\\ Sensitivity=\frac{TP_{NSR}}{TP_{NSR}+FN_{NSR}}\\ Specificity=\frac{TN_{NSR}}{TN_{NSR}+FP_{NSR}}\\ F1Score=\frac{TP_{NSR}}{TP_{NSR}+\left(0.5*\left(FP_{NSR}+FN_{NSR}\right)\right)} \end{array}$$

Let $Y_{j}^{i}$ be the data instances where *i* is the true class, *j* is the predicted class, and i,j∈{NSR,AF}. Consider the class AF signifying atrial fibrillation rhythms, and then, its outputs are defined as follows:

- $TP_{AF}=Y_{AF}^{AF}$, denotes data instances correctly classified as AF;
- $FP_{AF}=Y_{AF}^{NSR}$, denotes data instances incorrectly classified as AF;
- $FN_{AF}=Y_{NSR}^{AF}$, denotes data instances incorrectly classified as non-AF classes;
- $TN_{AF}=Y_{ij}$, denotes i,j≠AF, denotes data instances correctly classified as non-AF classes.

The aggregated scores across all 5 folds are summarized in Table 5, and exhibit a high AF classification performance. The true positive ($TP_{AF}$) rate is 96.90%, and the true negative $TN_{AF}$ rate is 95.13%. 

### 4.3. Model Evaluation on PPG Dataset

While evaluating the model on the PPG dataset, two scenarios are considered. In the first scenario, the weights of the pre-trained model were not updated through transfer learning. In the second scenario, the model was finetuned by retraining the PPG signals. 

In the first scenario, the model correctly classified 170 out of 192 samples of NSR, and 42 out of 54 samples as AF. The true positive ($TP_{AF}$) rate is 77.80%, and the true negative ($TN_{AF}$) rate is 88.54%. The measures reported in Table 6 serves as an initial benchmark test to gauge the performance of the ECG HRV trained on PPG data that have not been encountered during training or validation by the CNN model. 

In the second scenario, the learned weights of the model are updated by using 75% of UMass-DB for (60%) training and (15%) validation, with 25% for holdout testing, following the Stratified k-fold cross-validation with k = 4. By employing this approach, the intention is to adapt the weights of the pre-trained CNN model with 75% of the PPG data instances, test its performance on the remaining 25% of the untrained PPG data instances. This was applied four separate times, such that every instance is used for training, validation, and testing independently without data leakage between the training/validation and the testing sets. The aggregated testing performance is reported in Table 7, where the model makes predictions on all instances fairly.

After retraining, the average true positive ($TP_{AF}$) rate is 94.33% and the average true negative $TN_{AF}$ rate is 95.20%.

This performance is considerably high as the model classifies instances from a different input modality (PPG), when it was trained using only ECG signals. A marginal increase in performance is observed when transfer learning is implemented. Type I and type II errors were also observed, at a lower degree, resulting in AF false positives and AF false negatives, as shown by the results in Table 6 and Table 7. This indicates that the boundaries between the NSR and AF to a certain extent are not clearly distinct in both the ECG and PPG recordings. Factors, such as PPG sensor specifications, reliability, and quality, may contribute to the decreased classification measures compared to the training performance. It is to be noted that both the ECG training samples and the PPG samples were resampled using FFT at 50 Hz, 100 Hz, 128 Hz, 250 Hz and 360 Hz, corresponding to the different sampling rates of the original dataset recordings to see the differences in the achieved results. The conducted empirical experiments found that 50 Hz for all recordings yielded relatively similar performance when classifying PPG signals as ~128 Hz (the minimum sampling rate across all datasets).

### 4.4. Implementation and Testing

In addition to the validation conducted in Section 4.3, a prototype implementation was further developed and tested on live human subjects. The developed model was integrated within a health monitoring platform to test and ascertain its real-world performance. A smartphone application was designed to acquire PPG recordings, interface with the model, and retrieve predictions of AF from human subjects.

**Figure 6:**
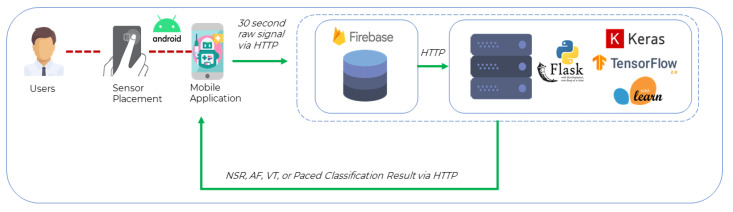
Implementation Architecture.

The system that implements the proposed CNN model presented in this work was realized by following the three-tier architecture for modularity, scalability, and testing. The model was deployed via a Python Flask [70] server with a Google Firestore [71] database on the same workstation. Figure 6 presents the smartphone application collecting the input from the sensor, i.e., raw PPG heart rhythm values and sending an HTTP POST request to the REST API server containing the recorded heart rhythm values. The smartphone application receives a response from the server (end-to-end response time ≈ 1.25 s) indicating whether the recording was NSR or AF.

The PPG signals from most variations of optical sensors available in general-purpose smartphones and wearable devices can be used in the classification of AF after applying the techniques of filtering, down-sampling, peak detection, PRV extraction as outlined in Section 3.3. The specifications of the particular sensor used in this implementation are listed in Table 8 and have a maximum frequency of 100.0 Hz. The sensor type is 65,572 and is manufactured by MAXIM. The heart rate monitor LED measures the magnitude of the red light reflected from an individual’s blood vessels at the measurement location, in the range of 0–350,000 (unitless). It operates on a 3.0 V to 5.5 V single supply voltage, with dimensions of 2.9 mm × 4.3 mm × 1.4 mm, and is integrated into portable or wearable devices. The devices used in the experiments were the Samsung S9, Samsung Note 8, and Samsung Note 9. M. Elgendi et al. [72] used Samsung 9th generation smartphones, the same ones used in this work.

The prototype implementation was successfully verified on the human subjects with the complete flow from signal acquisition to live AF classification following the same preprocessing techniques for filtering and resampling used for the UMass-DB PPG signals.

**Figure 7:**
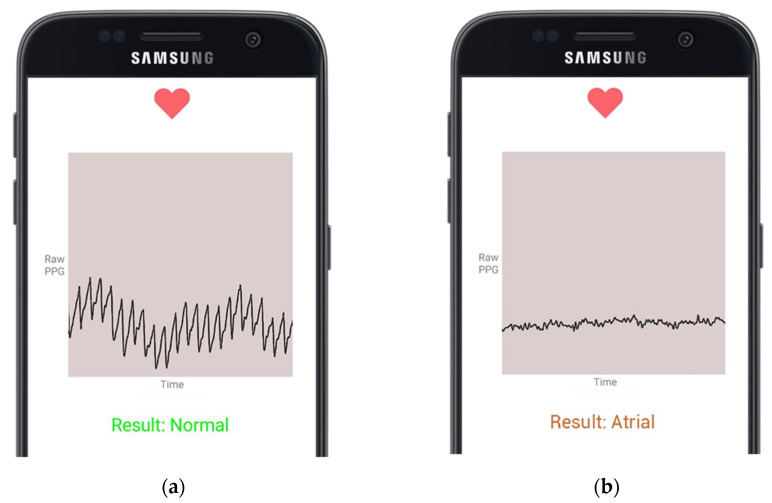
Cardiac arrythmia classifications presented on a smartphone application by the model using live PPG readings from the fingertip smartphone sensor; (**a**) presents NSR Classification; (**b**) presents AF Classification.

The human subjects were classified into healthy human subjects with no reported medical conditions, while the other was a heart patient from the Welcare Hospital Ernakulam, India. To record the heart rhythm, the subject is required to position their fingertip on the smartphone’s heart rate sensor. Upon the detection of the PPG input signal, the smartphone application initiates the PPG value acquisition process. The healthy subject continues to hold their fingertip in place for 30 s, and then, the signal is transmitted to the server. The model classified one of the short length heart rhythms obtained at rest as NSR, as shown in Figure 7a. The heart patients’ vitals are supervised through a bedside monitor by the doctor. Upon detecting an oncoming abnormality on the monitor, the patient is asked to place their finger on the smartphone and record a PPG signal. The result is shown in Figure 7b. The classification is saved in the cloud database under a specific entry for each subject, and the REST API server processes and responds to each acquired signal. This allows subjects and doctors to access historical records of the subject heart activity regularly.

**Figure 8:**
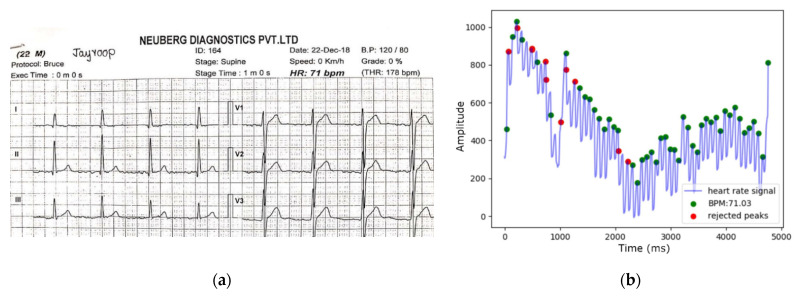
Measured ECG vs. estimated PPG BPM comparison. (**a**) Reference ECG-measured BPM; (**b**) PPG-derived BPM by peak detection algorithm.

The healthy subject underwent a Treadmill Stress Test in the clinical laboratory to observe the similarity in heartbeats and peak formations between an ECG and the PPG peak detection algorithm used in this work. The Treadmill Stress Test uses medical-grade multi-lead ECG to capture heart activity to measure cardiovascular health. A reference ECG signal was simultaneously collected to validate the PPG signal obtained from the smartphone sensors for the same 30 s. The resulting waveforms are shown in Figure 8a,b. Both Figure 8a,b estimate the same BPM, indicating potential consistency in the number of detected peaks.

## 5. Discussion

This study explored the efficiency of using convolutional neural networks to classify short-length heart rhythms using the concept of HRV-derived features to generalize AF representation across both the ECG and PPG modality. In this paper, the proposed model is compared and contrasted with similar works in the literature. The primary contributions of this research are highlighted in the following subsections. 

### 5.1. Comparison with Existing Works

Table 9 presents recent advances in the literature for short-length cardiac arrhythmia detection using one or more HRV features with applicability in portable devices.

Zhou et al. [17] employed a modified version of the Shannon entropy algorithm for AF detection by constructing symbolic sequences and probability distributions using ECG-based R-R intervals from the MIT AF database. This statistical approach was one of the first studies to discuss the possibility of deploying such approaches in portable devices. Islam et al. [73] presented a rhythm-based heartbeat normalization technique for improved ECG-based AF detection by measuring irregularities in a specified window of heartbeats. The datasets used for training and testing were the MIT-BIH AF database and MIT-BIH Arrhythmia, respectively. Cui et al. [18] proposed a similarity analysis and ensemble scheme that maps R-R intervals to binary symbolic sequences and compares the rank-frequencies to quantify the differences between AF and NSR using the ECG-based MIT-BIH AF database. Shashikumar et al. [74] presented one of the first and few works proposing cross-domain generalizability of cardiac arrhythmia models and used Bidirectional Recurrent Neural Network for AF detection from a single lead ECG. The researchers collected the ECG dataset from the University of Virginia Heart Station, United States, for training and collected the PPG dataset from the Emory Hospital and Grady Memorial Hospital, Atlanta, United States, for testing. They reported high classification performance for the cross-domain application using spectral features and R-R time series features with wavelet decomposition. Bashar et al. [75] utilized support vector machines on 30-s-long PPG signals for AF and NSR detection. They trained and tested on a custom-made PPG dataset and addressed noise saturation by using Butterworth filters. Tarniceriu et al. [76] implemented Markov models to detect AF and NSR by using R-R intervals as features and collected a dataset with a custom wearable prototype. Aliamiri et al. [77] employed an end-end deep learning PPG-based AF detection system that filters poor quality signals. They developed a convolution-recurrent hybrid model using waveform features on a custom-made PPG dataset that could effectively distinguish between AF and NSR. Tison et al. [78] conducted one of the first large-scale studies for passive AF detection using PPG-enabled smartwatches in collaboration with the University of California, San Francisco and Cardiogram. Cardiogram is an Apple watch application used to obtain heart rate data. The researchers used these collected data to implement a deep neural network with heuristic pretraining and R-R intervals as a feature set. Fallet et al. [79] utilized decision trees with waveform features and RR-intervals to classify AF and ventricular arrhythmia in 10-s-long PPG signals. The researchers created a PPG signal dataset from Lausanne University Hospital Switzerland and used a custom wearable prototype to test their results. Kwon et al. [80] employed a 1D CNN to process 30-s-long PPG signals to classify AF and NSR with a custom-made dataset.

**Table 9:** A comparison of recent works developed for CVD detection with machine learning and portable devices.

Author (Year)	Features	Approach	Modality	Accuracy (%)	Sensitivity (%)	Specificity (%)
This work	Temporal HRV	Convolutional Neural Networks	ECG; PPG	ECG = 95.50	ECG = 94.50	ECG = 96.00
				PPG = 95.10	PPG = 94.60	PPG = 95.20
Zhou et al. [17] (2015)	R-R intervals	Shannon Entropy	ECG	97.89	97.37	98.44
Cui et al. [18] (2017)	R-R intervals	Ensemble Model	ECG	97.78	97.04	96.97
Shashikumar et al. [74] (2018)	R-R Intervals and waveform features	Bidirectional Recurrent Neural Networks	ECG; PPG	ECG = 94.00 PPG = 95.00	-	ECG = 95.00 PPG = 100.00
Bashar et al. [75] (2018)	R-R intervals and waveform features	Support Vector Machines	PPG	91.16	-	-
Tarniceriu et al. [76] (2018)	R-R Intervals	Markov Model	PPG	-	98.45	99.13
Aliamiri et al. [77] (2018)	Waveform features	Convolutional Recurrent Neural Networks	PPG	98.19	-	-
Tison et al. [78] (2018)	R-R Intervals	Neural Network	PPG	-	98.00	90.20
Fallet et al. [79] (2019)	R-R intervals and waveform features	Decision Trees	PPG	95.00	92.90	96.20
Kwon et al. [80] (2019)	R-R intervals	Convolutional Neural Network	PPG	97.58	99.32	95.85

The performance measures obtained in this work are competitive with the works reported previously. The existing research has achieved successful results in the domain, however, has a few limitations that the proposed approach in this paper addresses. Firstly, the PPG datasets are not gold-standard and are not publicly accessible to reproducible and further testing. In this work, the reputed MIT-BIH datasets are utilized for implementing a cross-domain generalizable model. The input features of HRV captures a holistic representation of cardiac activity, as they are the most consistent medium of commonality between ECG and the PRV aspect of PPG signals. Secondly, existing models trained on ECG signals cannot be applied to predict PPG directly due to the differences in their morphology. In most of the works, ECG-based models can only work with portable devices having ECG sensors, and the PPG based-models require custom wearable prototypes or hospital settings, except in [78]. Thirdly, the developed models are not trained with multiple datasets or assessed on unseen data, lowering the likelihood of being applicable in non-ambulatory settings. Lastly, this work provides a supplementary approach, wherein the time-domain HRV representations are extracted from larger public datasets instead of raw signals, which extends the applicability to both ECG and PPG derived from clinical devices or consumer wearables.

### 5.2. Research Impact

This work presents a generalizable approach that has the potential for sensor agnostic CVD classification. The model is trained on data acquired from the source ECG modality and finetuned by updating the learned parameters using data from the target PPG modality. There were 15,434 instances from the ECG datasets of both NSR and AF for training the model, while there were only 192 total instances from the PPG dataset. Through the development of models with large cohorts of data in the related domain of ECG and the use of transfer learning, the issue of limited, gold-standard data accessibility from consumer wearable devices can be resolved. This can enable healthcare providers to leverage such devices in conjunction with cardiac arrhythmia classification models for non-ambulatory cardiovascular prognosis in the general population.

Smart healthcare platforms are holistic systems that enable disease prevention, monitoring, diagnosis, and treatment and connect patients with medical professionals. These are significant risk factors for the progression of CVD in patients. Repeated detection of any cardiovascular impairments as indicated by the AF in this work can prompt a clinical checkup, thereby allowing for early treatment and outcome improvement. A systematic survey by Majumder et al. [81] of 11 smartphone cardiac monitoring applications showed that the majority of them used simple, static heart rate threshold-based risk stratification. Furthermore, the existing solutions were not designed to be part of a monitoring system that can interface with clinicians but rather limited to the device only within the scope of the testing setting. Kakria et al. [82] proposed a real-time cardiac health monitoring system with a patient and doctor portal for effective monitoring using a custom Bluetooth wearable device and smartphone. However, medical alerts sent to patients and users lacked specificity, as any heartbeat above or below a threshold is flagged as abnormal. Moreover, there were no considerations for noise saturation or adaptability to signals other than PPG. In resource-constrained settings such as inexpensive fitness bands, extracting only the features necessary instead of complete raw signal samples can prove to be more efficient, as demonstrated in this work.

A possible limitation stems from the fact that there appears to be an overlap between the samples of each class. This could be due to the differences in resting heart rates across individuals, general fitness levels, and the influence of underlying health conditions. A direct approach to boost the model’s performance is to incorporate additional real ECG samples from more reputable datasets. Finally, spectral and non-linear HRV measures [83] can be added to the feature space to capture more robust representations of each class.

## 6. Conclusions

This work proposed a design and implementation of an explainable deep learning 1D-CNN model for use in smart healthcare systems with general-purpose devices such as smart wearables and smartphones. The 1D-CNN model classifies the NSR and AF from short length ECG or PPG signals using HRV features as inputs with the MIT-BIH ECG datasets.

The 1D-CNN model achieved overall classification performances with accuracy of 95.50%, sensitivity: 94.50%, specificity: 96.00%, F1-score: 93.40%, and AUC: 95.30% across a five-fold cross-validation approach. In comparison to other works in the literature, these performance measures are highly competitive and can be integrated into mobile health monitoring platforms with general-purpose devices. Thereby, the proposed approach is one of the first works to develop a cross-domain generalizable ECG-based model for deployment in smartphones and wearable devices.

Furthermore, the proposed methodology removes noise and motion artifacts from commercial PPG-sensors within a framework for health monitoring, thereby making early detection systems accessible for the general public. This approach brings to the forefront the applicability of ECG databases to enable machine learning to transform the PPG sensor readings from commercial devices. This can mitigate the issues of developing classification models that can only be used in controlled settings as well as increase the types of cardiac arrhythmia that can be observed from general-purpose devices and eliminate difficulties associated with creating custom PPG datasets for each study.

Subsequent research directions involve conducting a longitudinal study for exhaustive testing with users to attain additional empirical evidence supporting the real-world applicability of this approach, benchmarking the model against further gold-standard datasets, and extending the scope of the health monitoring framework.

## Figures and Tables

**Table 1:** A summary of the HRV feature characteristics used in this work.

Feature	Domain	Description
R-R interval	Time	Times between each successive heartbeat, measured from one normal or abnormal R peak/QRS to the next in milliseconds.
rMSSD	Time	Square root of the mean of the sum of the squares between adjacent R-R intervals.
SDRR	Time	Standard deviation of R-R intervals in milliseconds.
meanRR	Time	Average value of the R-R interval in milliseconds.
CVRR	Time	Coefficient of variation in R-R intervals.
CVSD	Time	Coefficient of variation between successive R-R interval differences.
medianRR	Time	Median value in R-R intervals in milliseconds.
madRR	Time	Median value of R-R interval deviation in milliseconds.
mcvRR	Time	Median value of the coefficient of variation.
RR20	Time	Number of pairs of adjacent R-R intervals differing by more than 20 milliseconds.
pRR20	Time	Count of RR20 over a total number of R-R intervals.
RR50	Time	Number of pairs of adjacent R-R intervals differing by more than 50 milliseconds.
pRR50	Time	Count of RR50 over the total number of R-R intervals.

**Table 2:** HRV Reference Ranges.

Feature	Range	Mean ± SD
RR interval (ms)	785–1160	926 ± 90
rMSSD (ms)	19–75	42 ± 15
SDRR (ms)	32–93	50 ± 16

**Table 3:** Total data Samples of NSR and AF classes after preprocessing.

Dataset	NSR	AF
ARR-DB(ECG)	2365	190
NSR-DB (ECG)	7736	
AF-DB (ECG)	83	5060
Total (ECG)	10,184	5250
UMass-DB (PPG)	192	54

**Table 4:** Summary of properties for the proposed CNN model.

Layers	Type	No. of Kernels	Kernel Size	Parameters
0–-1	Conv1D	256	3	Activation = ReLU, Strides = 1
1–2	BatchNormalization	–	–	–
2–3	Conv1D	128	3	Activation = ReLU, Strides = 1
3–4	BatchNormalization	–	–	-
4–5	Conv1D	64	3	Activation = ReLU, Strides = 1
5–6	BatchNormalization	–	–	–
6–7	Conv1D	32	3	Activation = ReLU, Strides = 1
7–8	BatchNormalization	–	–	–
8–9	Dropout	–	–	Rate = 0.2
9–10	MaxPooling1D	–	–	Pooling Size = 2
–	Flatten	–	–	–
10–11	Dense	8	–	Activation = ReLU
11–12	Dense	1	–	Activation = Sigmoid

**Table 5:** Aggregated classification metrics across five-folds expressed as mean and standard deviation.

Accuracy (%)	Sensitivity (%)	Specificity (%)	F1-Score (%)	AUC (%)
95.50 ± 0.2	94.50 ± 1.8	96.00 ± 0.7	93.36 ± 0.4	95.3 ± 0.5

**Table 6:** Performance measures of the ECG-trained model on the complete UMass-DB PPG signals before transfer learning.

Accuracy (%)	Sensitivity (%)	Specificity (%)	F1-Score (%)	AUC (%)
86.00	77.80	88.54	72.00	83.16

**Table 7:** Performance measures of the ECG-trained model on UMass-DB PPG signals after transfer learning folds expressed as mean and standard deviation.

Accuracy (%)	Sensitivity (%)	Specificity (%)	F1-Score (%)	AUC (%)
95.10 ± 2.9	94.6 ± 2.4	95.20 ± 6.5	89.34 ± 1.8	94.9 ± 4.10

**Table 8:** Sensor specifications for the smartphones used in this experiment.

Name	Vendor	Range	Voltage (V)	Type
HRMLED RED	MAXIM	0–350,000	3.0 V–5.5 V	65,572

## Data Availability

The ECG dataset adopted in this research is openly available in [Physionet] at https://doi.org/10.13026/C2F305, https://doi.org/10.13026/C2MW2D, and https://doi.org/10.13026/C2NK5R (accessed on 1 September 2021). The PPG dataset analyzed in this research is openly available in https://www.synapse.org/#!Synapse:syn23565056/wiki/608635 (accessed on 1 September 2021).

## References

[1] Cardiovascular Diseases. https://www.who.int/westernpacific/health-topics/cardiovascular-diseases.

[2] Cardiovascular Disease nhs.uk. 17 October 2017. https://www.nhs.uk/conditions/cardiovascular-disease/.

[3] Pereira T., Tran N., Gadhoumi K., Pelter M.M., Do D.H., Lee R.J., Colorado R., Meisel K., Hu X. (2020). Photoplethysmography based atrial fibrillation detection: A review. NPJ Digit. Med..

[4] Goldberger A.L., Goldberger Z.D., Shvilkin A., Goldberger A.L., Goldberger Z.D., Shvilkin A. (2018). Chapter 13-Sinus and Escape Rhythms. Goldberger’s Clinical Electrocardiography (Ninth Edition).

[5] Field M.E., Levine G.N. (2018). Chapter 35—Atrial Fibrillation. Cardiology Secrets.

[6] Electrocardiogram (ECG or EKG). www.heart.org. https://www.heart.org/en/health-topics/heart-attack/diagnosing-a-heart-attack/electrocardiogram-ecg-or-ekg.

[7] Sagahyroon A. Remote patients monitoring: Challenges. Proceedings of the 2017 IEEE 7th Annual Computing and Communication Workshop.

[8] Measuring the Heart—How Does ECG and PPG Work? iMotions, 21 March 2017. https://imotions.com/blog/measuring-the-heart-how-does-ecg-and-ppg-work/.

[9] MD M.C. Heart Rate Variability: A New Way to Track Well-Being. Harvard Health Blog. 22 November 2017. https://www.health.harvard.edu/blog/heart-rate-variability-new-way-track-well-2017112212789.

[10] (2019). Neurosky.com. http://neurosky.com/wp-content/uploads/2016/06/TOF-side-by-side-competitor-comparison.pdf.

[11] Paradkar N., Chowdhury S.R. Cardiac arrhythmia detection using photoplethysmography. Proceedings of the 2017 39th Annual International Conference of the IEEE Engineering in Medicine and Biology Society (EMBC).

[12] Koshy A.N., Sajeev J.K., Nerlekar N., Brown A.J., Rajakariar K., Zureik M., Wong M.C., Roberts L., Street M., Cooke J. (2018). Utility of photoplethysmography for heart rate estimation among inpatients. Intern. Med. J..

[13] Millán C.A., Girón N.A., Lopez D.M. (2020). Analysis of Relevant Features from Photoplethysmographic Signals for Atrial Fibrillation Classification. Int. J. Environ. Res. Public Health.

[14] Aschbacher K., Yilmaz D., Kerem Y., Crawford S., Benaron D., Liu J., Eaton M., Tison G.H., Olgin J.E., Li Y. (2020). Atrial fibrillation detection from raw photoplethysmography waveforms: A deep learning application. Hear. Rhythm O2.

[15] Alian A.A., Shelley K.H. (2014). Photoplethysmography. Best Pract. Res. Clin. Anaesthesiol..

[16] Charlton P., Bonnici T., Tarassenko L., Alastruey J., Clifton D.A., Beale R., Watkinson P. (2017). Extraction of respiratory signals from the electrocardiogram and photoplethysmogram: Technical and physiological determinants. Physiol. Meas..

[17] Zhou X., Ding H., Wu W., Zhang Y. (2015). A Real-Time Atrial Fibrillation Detection Algorithm Based on the Instantaneous State of Heart Rate. PLoS ONE.

[18] Cui X., Chang E., Yang W.-H., Jiang B.C., Yang A.C., Peng C.-K. (2017). Automated Detection of Paroxysmal Atrial Fibrillation Using an Information-Based Similarity Approach. Entropy.

[19] Dash S., Chon K.H., Lu S., Raeder E.A. (2009). Automatic real time detection of atrial fibrillation. Ann. Biomed. Eng..

[20] Tateno K., Glass L. (2001). Automatic detection of atrial fibrillation using the coefficient of variation and density histogram of NN and NN intervals. Med. Biol. Eng. Comput..

[21] Hagiwara Y., Fujita H., Oh S.L., Tan J.H., Tan R.S., Ciaccio E.J., Acharya U.R. (2018). Computer-aided diagnosis of atrial fibrillation based on ECG Signals: A review. Inf. Sci..

[22] Yıldırım Ö., Pławiak P., Tan R.S., Acharya U.R. (2018). Arrhythmia detection using deep convolutional neural network with long duration ECG signals. Comput. Biol. Med..

[23] Acharya U.R., Oh S.L., Hagiwara Y., Tan J.H., Adam M., Gertych A., Tan R.S. (2017). A deep convolutional neural network model to classify heartbeats. Comput. Biol. Med..

[24] Kiranyaz S., Ince T., Gabbouj M. (2017). Personalized Monitoring and Advance Warning System for Cardiac Arrhythmias. Sci. Rep..

[25] Ramos G., Alfaras M., Gamboa H. Real-Time Approach to HRV Analysis. Proceedings of the 11th International Joint Conference on Biomedical Engineering Systems and Technologies.

[26] Bent B., Goldstein B.A., Kibbe W.A., Dunn J.P. (2020). Investigating sources of inaccuracy in wearable optical heart rate sensors. NPJ Digit. Med..

[27] Elgendi M. (2012). On the Analysis of Fingertip Photoplethysmogram Signals. Curr. Cardiol. Rev..

[28] Malik M., Camm A.J., Bigger J.T., Breithardt G., Cerutti S., Cohen R.J., Coumel P., Fallen E.L., Kennedy H.L., Kleiger R.E. (1996). Heart rate varia-bility. Standards of measurement, physiological interpretation, clinical use. Eur. Heart J..

[29] Smith A.-L., Owen H., Reynolds K. (2013). Heart rate variability indices for very short-term (30 beat) analysis. Part 1: Survey and toolbox. J. Clin. Monit..

[30] Lu S., Zhao H., Ju K., Shin K., Lee M., Shelley K., Chon K.H. (2007). Can Photoplethysmography Variability Serve as an Alternative Approach to Obtain Heart Rate Variability Information?. J. Clin. Monit..

[31] Jeyhani V., Mahdiani S., Peltokangas M., Vehkaoja A. Comparison of HRV parameters derived from photoplethys-mography and electrocar-diography signals. Proceedings of the 2015 37th annual international conference of the IEEE engineering in medicine and biology society (EMBC).

[32] Shaffer F., Ginsberg J.P. (2017). An Overview of Heart Rate Variability Metrics and Norms. Front. Public Health.

[33] O’Neal W.T., Chen L., Nazarian S., Soliman E.Z. (2016). Reference ranges for short-term heart rate variability measures in individuals free of cardiovascular disease: The Multi-Ethnic Study of Atherosclerosis (MESA). J. Electrocardiol..

[34] Moody G.B., Mark R.G. (2001). The impact of the MIT-BIH Arrhythmia Database. IEEE Eng. Med. Biol. Mag..

[35] Goldberger A.L., Amaral L.A.N., Glass L., Hausdorff J.M., Ivanov P.C., Mark R.G., Mietus J.E., Moody G.B., Peng C.-K., Stanley H.E. (2000). PhysioBank, PhysioToolkit, PhysioNet. Circulation.

[36] American Association of Medical Instrumentation (2013). ANSI/AAMI EC57: 2012—Testing and Reporting Performance Results of Cardiac Rhythm and ST Segment Measurement Algorithms. American National Standard.

[37] Teijeiro T., Felix P., Presedo J.M.R., Castro D. (2016). Heartbeat classification using abstract features from the abductive interpretation of the ECG. IEEE J. Biomed. Health Inform..

[38] Proenca T., Carvalho M.M., Pinto R.A., Resende C., Grilo P., Torres S., Paiva M., Lebreiro A., Campelo M., Rema J. (2020). Supraventricular ectopic activity as a predictor of atrial fibrillation—what we didn’t see 10 years ago. Eur. Heart J..

[39] Sörnmo L., Laguna P. (2005). Bioelectrical Signal Processing in Cardiac and Neurological Applications.

[40] Rajoub B. (2020). Biomedical Signal Processing and Artificial Intelligence in Healthcare.

[41] Han D., Bashar S.K., Mohagheghian F., Ding E., Whitcomb C., McManus D.D., Chon K.H. (2020). Premature Atrial and Ventricular Contraction Detection using Photoplethysmographic Data from a Smartwatch. Sensors.

[42] Bashar S.K., Han D., Hajeb-Mohammadalipour S., Ding E., Whitcomb C., McManus D.D., Chon K.H. (2019). Atrial Fibrillation Detection from Wrist Photoplethysmography Signals Using Smartwatches. Sci. Rep..

[43] Binici Z., Intzilakis T., Nielsen O.W., Køber L., Sajadieh A. (2010). Excessive Supraventricular Ectopic Activity and Increased Risk of Atrial Fibrillation and Stroke. Circulation.

[44] Ding E.Y., Han D., Whitcomb C., Bashar S.K., Adaramola O., Soni A., Saczynski J., Fitzgibbons T.P., Moonis M., Lubitz S.A. (2019). Accuracy and Usability of a Novel Algorithm for Detection of Irregular Pulse Using a Smartwatch Among Older Adults: Observational Study. JMIR Cardio.

[45] Ebrahimi Z., Loni M., Daneshtalab M., Gharehbaghi A. (2020). A review on deep learning methods for ECG arrhythmia classification. Expert Syst. Appl. X.

[46] Elgendi M., Jonkman M., Boer F.D. Frequency bands effects on QRS detection. Proceedings of the BIOSIGNALS 2010—Proceedings of the 3rd International Conference on Bioinpsired Systems and Signal Processing.

[47] Pan J., Tompkins W.J. (1985). A Real-Time QRS Detection Algorithm. IEEE Trans. Biomed. Eng..

[48] Li B.N., Dong M.C., Vai M.I. (2010). On an automatic delineator for arterial blood pressure waveforms. Biomed. Signal Process. Control.

[49] Elgendi M., Norton I., Brearley M., Abbott D., Schuurmans D. (2013). Systolic Peak Detection in Acceleration Photoplethysmograms Measured from Emergency Responders in Tropical Conditions. PLoS ONE.

[50] Billauer E. Peakdet: Peak Detection Using MATLAB. http://billauer.co.il/peakdet.html.

[51] Zong W., Heldt T., Moody G., Mark R. An open-source algorithm to detect onset of arterial blood pressure pulses. Proceedings of the Computers in Cardiology.

[52] Mahdiani S., Jeyhani V., Peltokangas M., Vehkaoja A. Is 50 Hz High Enough ECG Sampling Frequency for Accurate HRV Analysis? The work was partially funded by the Finnish Funding Agency for Technology and Innovation (TEKES) as a part of project VitalSens (decision ID 40103/14). Proceedings of the 2015 37th Annual International Conference of the IEEE Engineering in Medicine and Biology Society (EMBC).

[53] Béres S., Hejjel L. (2021). The minimal sampling frequency of the photoplethysmogram for accurate pulse rate variability parameters in healthy volunteers. Biomed. Signal Process. Control.

[54] LeCun Y., Bengio Y. (1998). Convolutional networks for images, speech, time series. The handbook of Brain Theory and Neural Networks.

[55] Goodfellow Y., Bengio A. (2016). Courville, Deep Learning.

[56] Agarap A.F. (2019). Deep Learning using Rectified Linear Units (ReLU). arXiv.

[57] Han J., Moraga C. (1995). The influence of the sigmoid function parameters on the speed of back-propagation learning. From Natural to Artificial Neural Computation.

[58] Nielsen M.A. (2015). Neural Networks and Deep Learning.

[59] Ioffe S., Szegedy C. (2015). Batch Normalization: Accelerating Deep Network Training by Reducing Internal Covariate Shift. arXiv.

[60] Srivastava N., Hinton G., Krizhevsky A., Sutskever I., Salakhutdinov R. (2014). Dropout: A Simple Way to Prevent Neural Networks from Over-fitting. J. Mach. Learn. Res..

[61] Masters D., Luschi C. (2018). Revisiting Small Batch Training for Deep Neural Networks. arXiv.

[62] Kingma D.P., Ba J. (2017). Adam: A Method for Stochastic Optimization. arXiv.

[63] Kohavi R. A study of cross-validation and bootstrap for accuracy estimation and model selection. Proceedings of the 14th International Joint Conference on Artificial Intelligence—Volume 2.

[64] Chollet F. (2015). Keras. https://github.com/fchollet/keras.

[65] Abadi M., Barham P., Chen J., Chen Z., Davis A., Dean J., Devin M., Ghemawat S., Irving G., Isard M. TensorFlow: A System for Large-Scale Machine Learning. Proceedings of the Proceedings of the 12th USENIX Conference on Operating Systems Design and Implementation.

[66] Virtanen P., Gommers R., Oliphant T.E., Haberland M., Reddy T., Cournapeau D., Burovski E., Peterson P., Weckesser W., Bright J. (2020). SciPy 1.0: Fundamental Algorithms for Scientific Computing in Python. Nat Methods.

[67] Makowski D. (2016). NeuroKit: A Python Toolbox for Statistics and Neurophysiological Signal Processing (EEG, EDA, ECG, EMG...).

[68] Wong H.B., Lim G.H. (2011). Measures of Diagnostic Accuracy: Sensitivity, Specificity, PPV and NPV. Proc. Singap. Health.

[69] Tharwat A. (2018). Classification assessment methods. Appl. Comput. Inform..

[70] Welcome to Flask—Flask Documentation (1.1.x). https://flask.palletsprojects.com/en/1.1.x/.

[71] Cloud Firestore|Firebase. https://firebase.google.com/docs/firestore.

[72] Elgendi M., Fletcher R., Liang Y., Howard N., Lovell N.H., Abbott D., Lim K., Ward R. (2019). The use of photoplethysmography for assessing hypertension. NPJ Digit. Med..

[73] Islam S., Ammour N., Alajlan N., Aboalsamh H. (2016). Rhythm-based heartbeat duration normalization for atrial fibrillation detection. Comput. Biol. Med..

[74] Shashikumar S.P., Shah A.J., Clifford G.D., Nemati S. (2018). Detection of Paroxysmal Atrial Fibrillation using Attention-based Bidirectional Recurrent Neural Networks. arXiv.

[75] Bashar S.K., Han D., Soni A., McManus D.D., Chon K.H. Developing a novel noise artifact detection algorithm for smartphone PPG signals: Preliminary results. Proceedings of the 2018 IEEE EMBS International Conference on Biomedical Health Informatics (BHI).

[76] Tarniceriu A., Harju J., Yousefi Z.R., Vehkaoja A., Parak J., Yli-Hankala A., Korhonen I. The Accuracy of Atrial Fibrillation Detection from Wrist Photoplethysmography. A Study on Post-Operative Patients. Proceedings of the 2018 40th Annual International Conference of the IEEE Engineering in Medicine and Biology Society (EMBC).

[77] Aliamiri A., Shen Y. Deep learning based atrial fibrillation detection using wearable photoplethysmography sensor. Proceedings of the 2018 IEEE EMBS International Conference on Biomedical Health Informatics (BHI).

[78] Tison G., Sanchez J.M., Ballinger B., Singh A., Olgin J.E., Pletcher M.J., Vittinghoff E., Lee E.S., Fan S.M., Gladstone R.A. (2018). Passive Detection of Atrial Fibrillation Using a Commercially Available Smartwatch. JAMA Cardiol..

[79] Fallet S., Lemay M., Renevey P., Leupi C., Pruvot E., Vesin J.-M. (2018). Can one detect atrial fibrillation using a wrist-type photoplethysmographic device?. Med Biol. Eng. Comput..

[80] Kwon S., Hong J., Choi E.-K., Lee E., Hostallero D.E., Kang W.J., Lee B., Jeong E.-R., Koo B.-K., Oh S. (2019). Deep Learning Approaches to Detect Atrial Fibrillation Using Photoplethysmographic Signals: Algorithms Development Study. JMIR mHealth uHealth.

[81] Majumder S., Deen M.J. (2019). Smartphone Sensors for Health Monitoring and Diagnosis. Sensors.

[82] Kakria P., Tripathi N.K., Kitipawang P. (2015). A Real-Time Health Monitoring System for Remote Cardiac Patients Using Smartphone and Wearable Sensors. Int. J. Telemed. Appl..

[83] Huikuri H.V., Perkiömäki J.S., Maestri R., Pinna G.D. (2009). Clinical impact of evaluation of cardiovascular control by novel methods of heart rate dynamics. Philos. Trans. R. Soc. A Math. Phys. Eng. Sci..

